# Cross-sectional prospective survey to study indication-based usage of antimicrobials in animals: Results of use in cattle

**DOI:** 10.1186/1746-6148-4-15

**Published:** 2008-04-14

**Authors:** Katariina Thomson, Merja Rantala, Maria Hautala, Satu Pyörälä, Liisa Kaartinen

**Affiliations:** 1 University of Helsinki, Faculty of Veterinary Medicine, Department of Equine and Small Animal Science, P.O. Box 57, University of Helsinki, FI-00014 Helsinki, Finland; 2 National Center for Epidemiology, Gyáli út 2-6, H-1092 Budapest, Hungary; 3 University of Helsinki, Faculty of Veterinary Medicine, Department of Production Animal Medicine, Pohjoinen pikatie 800, FI-04920 Saarentaus, Finland; 4 Finnish Food Safety Authority Evira, Mustialankatu 3, FI-00790 Helsinki, Finland

## Abstract

**Background:**

Indication-based data on the use of antimicrobials in animals were collected using a prospective cross-sectional survey, similarly as for surveys carried out in human medicine, but adapting the questionnaire to include veterinary-specific issues. The participating veterinarians were randomly selected from a sample population of practising veterinarians. The sampling was stratified to take into account the proportions of different types of veterinary practice in the country. All patients consulting the veterinary practice during a 1-week period were included in the study and veterinarians returned a completed questionnaire for each patient receiving antimicrobial treatment. As cattle received most of the treatments, results from the survey are given using cattle as an example species.

**Results:**

The survey was sent to 681 veterinarians, of whom 262 (39%) responded. In total 2850 questionnaires were completed. The largest quantities of antimicrobials, measured in kilograms, were used for cattle, followed by pigs, dogs and horses. The species that were treated most were cattle (n = 1308), dogs (n = 989) and cats (n = 311). For cattle, the most common reason for treatment was acute mastitis (52%), followed by dry-cow therapy (21%), subclinical mastitis (6%) and treatment for acute enteritis (4%). The remaining treatments covered 17% of cattle patients and 15 different indications. For acute mastitis, parenteral or intramammary treatment was used in 36% and 34% of the cases, respectively. The remaining 30% received both treatments simultaneously. Of the parenteral treatments (n = 459), benzyl penicillin was used in 83% of the treated animals (n = 379), while fluoroquinolones were used in 49 cases (11%). Of the 433 cows receiving intramammary treatment, ampicillin combined with cloxacillin was most commonly used (n = 157; 36%), followed by cephalexin+streptomycin (n = 113; 26%).

**Conclusion:**

This cross-sectional prospective survey provided a useful method for the collection of information on the indication-based use of antimicrobials in different animal species. Cattle were the most commonly treated animal species during the study period. The most common indication for antimicrobial use in cows was mastitis. Benzyl penicillin was the drug most frequently used for the treatment of mastitis, which seems appropriate according to the national guidelines on the use of antimicrobials in cattle in Finland.

## Background

The lack of species-specific data on antimicrobial use has been recognised as a major problem when trying to identify the relationship between use and the development of resistance [1,2]. Several countries publish annual data on the overall use of antimicrobials in the treatment of animal diseases [3-7]. However, those data only provide consumption figures on a general level. More detailed information is needed in order to understand better the relationship between consumption; indicating the general exposure of microbial flora to antimicrobials, and resistance. Information on the use of antimicrobials in different animal species on an indication basis would also make it possible to evaluate how recommendations and prudent use guidelines for antimicrobial use are being followed.

Some studies have been carried out to collect more specific information on the use of antimicrobials in animals. For example, Chauvin et al. (2005) [8] described continuous monitoring of antimicrobial use in French poultry production. Busani et al. (2004) [9] conducted a telephone survey to evaluate how Italian veterinarians followed guidelines for the judicious use of antimicrobials. Some reports exist from Canada and the United States on data collected from dairy farms [10-12] and data based on wholesaler statistics from Norway and Sweden estimating antimicrobial usage for mastitis are available [13]. To the best of our knowledge, only Denmark collects detailed data on a continuous basis, recording information about the use of antimicrobials for different food-producing animals [4,14,15].

A cross-sectional prospective survey was previously successfully carried out to investigate indication-based use of antimicrobials in humans in Finland [16]. Our aim was to collect data using a similar method, to analyse the distribution of indication-based antimicrobial use among different animal species and to evaluate how the recommendations for antimicrobial use are being followed by veterinarians. The questionnaire was modified to include veterinary-specific issues (Table 1). Here we report the results as a descriptive analysis of indication-based use of antimicrobials in cattle in Finland.

**Table 1:** Information collected in the survey on antimicrobial use and variables entered into the database

**Data recoded**	**Type of variable**	**Description**
1. Animal species	Coded	Dog, cat, horse, cattle, pig, fur animals, fish, other
2. Type of visit	Coded	Normal daytime visit, on-call visit or prescription by phone
3. Main diagnosis	Coded	18 pre-coded alternatives and possibility to give own diagnosis if something else
4. Duration of clinical signs	Coded	0–3, 4–7, 8–14 days or longer
5. Clinical examination or diagnostic tests carried out	Coded	8 pre-coded alternatives and possibility to describe other tests or procedures
6. Antimicrobial drug administered by veterinarian	Text	Product name and strength; amount given
7. Antimicrobial drug given to the owner to continue the treatment (per oral or injectable)	Text	Product name and strength; duration of treatment
8. Antimicrobial drug given to the owner to continue the treatment (local treatment, including intramammaries)	Text	Product name and strength; duration of treatment
9. Was the choice of product used affected by allergy, other disease, owner's wishes, recurrent or chronic infection or something else?	Coded	
10. Was this the first visit or a follow-up visit?	Coded	

## Results

### Response rate and numbers of animals treated

A total of 262 of 681 (38%) veterinarians responded to the questionnaire (Table 2). The representativeness of respondents was tested for type of practice, geographical area, gender, year of graduation, degree and field of specialisation. Those who responded represented the intended sample well. The only statistically significant difference was noted in gender: female veterinarians responded more actively than males (p < 0.05).

**Table 2:** Background information on the veterinary practitioners

**Type of practice among those participating in the survey**	**Number of members (% of all members in practice)**	**Sample size (% of members in the named group)**	**Number of respondents (% of sampled practitioners in the group)**	**% of all respondents (n = 262)**
Community/county veterinarian in countryside	233 (27%)	176 (76%)	73 (41%)	27.9
Community/county veterinarian working in city	119 (14%)	91 (76%)	35 (38%)	13.4
Community/county head of health services	13 (2%)	13 (100%)	9 (69%)	3.4
Government or Helsinki University	80 (9%)	42 (53%)	19 (45%)	7.3
Private practitioner	180 (21%)	132 (73%)	48 (36%)	18.3
Veterinarians with non-permanent positions	141 (16%)	136 (96%)	47 (35%)	17.9
Student with right to practice the profession	92 (11%)	91 (99%)	31 (34%)	11.8
**Total**	**858**	**681 (79%)**	**262 (38%)**	**100**

**Figure 1:**
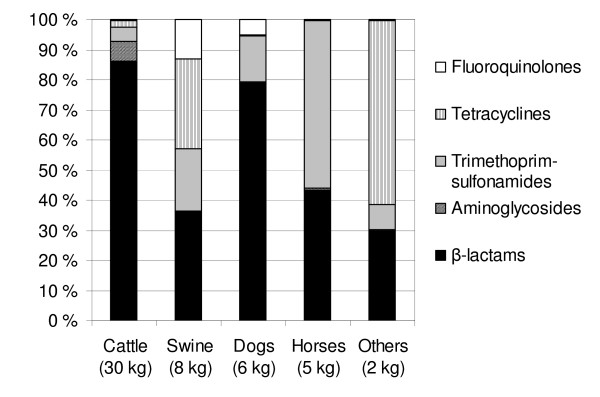
**Distribution of antimicrobial substances used for different animal species during the one-week study period**. 'Others' includes cats, fur animals, rabbits, small rodents and reptiles.

During the one-week survey, the responding veterinarians used 51 kg of antimicrobials, most of this for cattle (30 kg; 59%), pigs (8 kg; 16%), dogs (6 kg; 12%) and horses (5 kg; 10%). The distribution of the different groups of antimicrobials for each species is illustrated in Figure 1.

The 262 veterinarians that responded filled out questionnaires for a total of 2850 animals. Cattle were most commonly treated (n = 1308; 46%), followed by dogs (n = 989; 35%) and cats (n = 311; 11%). Six veterinarians treated solely cattle and were responsible for the treatments of 3.8% of all bovine cases. The remaining veterinarians also treated other animal species. As cattle were the species receiving most treatments, results from the survey are given using cattle as an example species.

### Antimicrobial usage in cattle

During the study period, a total of 1308 cattle were treated with antimicrobials. The most common diagnosis was acute mastitis (n = 686; 52%), followed by dry-cow therapy (n = 271; 21%), subclinical mastitis (n = 78; 6%) and acute enteritis (n = 49; 4%). The remaining treatments (n = 224; 17%) were distributed among 15 indications with the number of animals treated falling below 40 in each indication (abscess, septic arthritis, retained placenta etc.). The proportions of different antimicrobials used in cattle are presented in Figure 1. Of a total of 1035 treatments for mastitis, one third (n = 337) were prescribed by telephone to pharmacies. In dry-cow therapy almost half of the prescriptions were of this type (139 of 271) and all were intramammary treatments. For 10 cows (4%) an injectable preparation was prescribed by phone and seven of these were from the same herd.

### Acute mastitis

**Figure 2:**
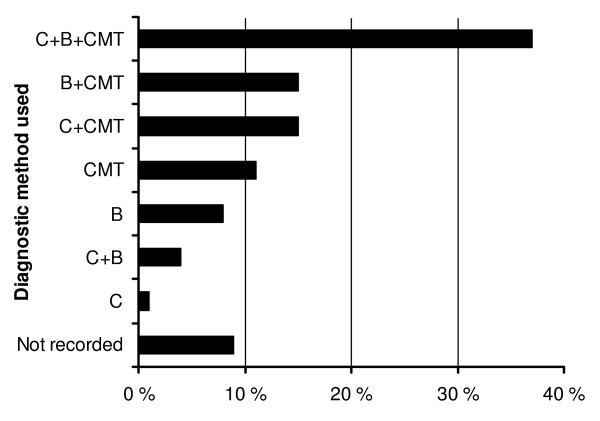
**Methods applied by veterinarians to diagnose acute bovine mastitis**. C = clinical signs, B = bacteriological culture and sensitivity testing, CMT = California Mastitis Test, not recorded = no diagnostics were used or none recorded.

Most veterinarians (37%) based the diagnosis of acute mastitis on clinical signs, results from the California Mastitis Test (CMT) and bacteriological culture (Figure 2). Most of the cows (482 of 686; 70%) had shown clinical signs of mastitis for 0–3 days before treatment. Eighteen percent (n = 122) showed some signs for 4–7 days before any treatment was initiated. The treatment regimen was documented for 679 animals (99%). Either parenteral treatment by intramuscular injection (n = 246; 36%) or intramammary treatment (n = 234; 34%) was used. A combination of parenteral and intramammary treatment was used in the remaining cases (n = 199). Thirteen animals (2%) were simultaneously treated with 2 or 3 parenteral preparations.

The most commonly used antimicrobial for parenteral treatment was benzyl penicillin (n = 379; 83%). Fluoroquinolones (enrofloxacin or danofloxacin) were used in 49 (11%) cases. Some cows were also treated with trimethoprim-sulfonamides (n = 20), oxytetracycline (n = 7) or spiramycin (n = 4). Of the 433 cows that received intramammary treatment, a product containing ampicillin and cloxacillin was the most commonly used (n = 157; 36%). Another frequently used product contained cephalexin and streptomycin (n = 113; 26%).

The duration of treatment was recorded for 413 cows treated parenterally and for 401 cows with intramammary treatment. In both groups the prescribed treatment length (median) was 4 days (43% and 41% respectively, range in both 1–8 days).

### Subclinical mastitis

A total of 78 cows were diagnosed as having subclinical mastitis during the study period. In 55 cows (71%), bacterial culture was used for diagnosis. Most of the cows (74%) were treated with intramammary preparations alone. As for acute mastitis, the most commonly used intramammaries were those containing cephalexin and streptomycin (35%), followed by ampicillin and cloxacillin (18%). The prescribed duration of treatment ranged from 1 to 8 days, and the median duration was 4 days.

### Dry cow therapy

Cows were selected for dry cow therapy on the basis of bacterial culture of milk in 41% (110 out of 271) of the cases. In 50 more cases, the veterinarian reported the use of the CMT as a diagnostic tool.

The vast majority of the dry cow therapies were intramammary treatments (94%). Of the 254 intramammary dry cow treatments, either cloxacillin alone or combined with ampicillin (n = 127; 50%), or a β-lactam combined with an aminoglycoside (n = 109; 43%) was used. A little over half of the medications were dispensed via telephone prescriptions (n = 139; 55%).

## Discussion

This cross-sectional prospective survey, using a method adapted from human medicine [16], represented a useful method for collecting information on the indication-based use of antimicrobials in animals. The response rate of almost 40% can be considered reasonably high, taking into account that completion of a survey questionnaire represents an additional burden for busy practitioners. The respondents represented the original sample very well (Table 2), which shows that the weighted stratification was successful.

The questionnaire functioned well. However, we identified a few items that could be added to obtain more information. For example, the weight of the animal should be recorded to calculate the actual dose used for the treatment. Information on the results from bacteriological cultures would improve the evaluation of how the guidelines were followed by the veterinarians. Antimicrobial drugs administered or dispensed (points 6–8 in the questionnaire, Table 1) could be combined in order to simplify the form. Having an electronic form could make the survey more user-friendly and also facilitate compiling the information supplied.

Mastitis was by far the most common indication for the use of antimicrobial agents in our study, as 79% of all the cows were treated for mastitis. This is consistent with previous studies [12,13,17]. Acute mastitis was most commonly treated within the first three days following the onset of clinical signs, but sometimes a cow was treated no earlier than 4–7 days after the appearance of the clinical signs. The urgency of the treatment should depend on the severity of the infection. In a recent study, a 24-h delay was found not to affect the therapy response of clinical mastitis with mild to moderate clinical signs [18]. The median length of treatment prescribed by the veterinarians was 4 days for all routes of administration. This is in line with the recommendations, which suggest a course of treatment of 3–5 days, independent of the treatment regimen used [19]. However, performing follow-up on owner compliance to complete the started or prescribed course of treatment was beyond the scope of this study.

The recommended practice in acute mastitis is to perform a clinical examination and the CMT and to take a milk sample for bacterial culture. Empiric treatment is initiated and directed towards the most probable causative pathogen [19]. This survey showed that only 37% of veterinarians used the recommended methods to make treatment decisions (Figure 2). In the majority of cases (73%) of subclinical mastitis, veterinarians used bacteriological diagnosis to target the treatment, as treatment could be postponed until results from the bacteriological culture were available. Dry cow therapy was based on bacteriological results in only 41% of cases. Bacteriological diagnosis as a basis for mastitis treatment could be used more frequently than was indicated in this survey.

National guidelines for the use of antimicrobials in animals have been provided by the Finnish Veterinary Antimicrobial Advisory Group of the Ministry of Agriculture and Forestry [20]. In Finland, half of acute mastitis cases are caused by staphylococci and streptococci [21]. The national guidelines suggest that acute mastitis caused by streptococci and β-lactamase-negative staphylococci should be treated with benzyl penicillin. In our survey, systemic treatment with β-lactams, mainly benzyl penicillin, was used for acute mastitis in the majority of cases. Drug selection thus accorded well with the recommendations, but the route of administration did not. Systemic treatment of acute mastitis is still common practice in all Nordic countries [13], despite the lack of evidence of its superiority over intramammary treatment [19]. About 30% of acute mastitis was treated using concomitant parenteral and intramammary treatment. Better efficacy of this regimen could be expected for deep infections such as mastitis caused by *Staphylococcus aureus*, but in mild to moderate clinical mastitis intramammary treatment is mostly sufficient [19,22]. Consumption of antimicrobials is much higher if parenteral or combination treatment is used for the routine treatment of mastitis.

Fluoroquinolones, which are the drugs of choice for severe coliform mastitis, were used in 11% of parenteral treatments for acute mastitis. About twelve percent of acute mastitis cases in Finland are caused by coliforms [21], so the use of fluoroquinolones reflected this proportion if targeted at these cases. The use of fluoroquinolones, particularly in food-producing animals, should be kept to a minimum to avoid the emergence of resistance [23]. Cattle are food-producing animals, which limits the variety of drugs that are approved for the treatment of infectious diseases. In Finland, the use of antimicrobial agents is further restricted by national regulations so that, for example, the use of 3rd and 4th generation cephalosporins in cattle is not permitted.

In order to further evaluate how closely the guidelines were followed when treating an individual patient, the results of bacteriological cultures would have been needed.

## Conclusion

This cross-sectional prospective survey proved useful in collecting indication-based data on the use of antimicrobials in animals. The response rate of veterinarians was 39%, which can be considered satisfactory. The most common indication for antimicrobial use was mastitis in cattle. Benzyl penicillin was the most commonly used drug. The first-line treatment of mastitis was in accordance with the national guidelines, except for the route of administration. The use of bacteriological diagnosis to target treatment was sub-optimal.

## Methods

### Prospective cross-sectional survey

The data were collected using a prospective cross-sectional survey according to the principles of surveys carried out in human medicine [16]. The questionnaire in our survey was modified to take into account the veterinary-specific issues.

In Finland 96% of the veterinarians are members of the Finnish Veterinary Association. The sample population comprised practising veterinarians (n = 858) and by using the computerised register of members, a random sampling of veterinarians (n = 681) was carried out. Non-practising veterinarians were excluded. The sampling was weighted in order to take into account the proportions of different types of veterinary practice to obtain a representative sample of each stratum (Table 2).

A letter explaining the study protocol and a sheet for collection of background information was sent to each participating practitioner with 20 questionnaire sheets. More could be copied if needed. The following data were collected from each practitioner as background information: geographical area (province), gender, year of graduation, degree and field of specialisation. The background sheet was numbered, indicating the stratum to which the veterinarian belonged.

Veterinarians were asked to complete one questionnaire sheet for every animal that received antimicrobial treatment during the 7-day (Monday to Sunday, one week in May) study period. The contents of the questionnaire are listed in Table 1. If the same treatment was carried out for several animals of the same species at one occasion, only one sheet was filled out, supplemented with the number of animals treated. When analysing the data, treatment for each animal was counted separately. The duration of clinical signs was estimated by the veterinarian according to the history provided by the animal owners. The duration of the treatment was the same as the prescribed length of the treatment.

Veterinarians had a possibility to reply anonymously. To motivate the practitioners to return the questionnaires, every respondent received feedback about the results of the study, and three travel gift certificates were also raffled between those who responded. Contact details were collected for this purpose.

### Data analysis

The distribution of the different practice types was compared with that of the source population. The following characteristics of the respondents were analysed: type of practice (stratum), geographical area, gender, year of graduation, degree and field of specialisation. The representativeness of the respondents compared with the original sample was analysed with a X^2^-test. The level of significance was set at p ≤ 0.05.

Precoded information from the questionnaires was introduced into a database in ASCII-format. Text fields describing the antimicrobial products were coded by taking into consideration whether the preparation used was a human or a veterinary product, the formulation and which group of antimicrobial substances it belonged to. A descriptive analysis of the distributions of antimicrobial use in different indications was performed. Both SAS (version 9.1, SAS Institute Inc, Cary, NC) and Microsoft Excel (version 10, Microsoft Corporation, Redmond, WA, USA) programmes were used for data analysis.

## Authors' contributions

KT analysed the data and drafted the manuscript. MR designed and carried out the survey, analysed the data and drafted the manuscript. MH analysed the data. SP drafted the manuscript. LK drafted the manuscript. All authors read, commented on and approved the final manuscript
